# The role of the media in the coverage of childhood vaccination in children under two years of age in Peru, ENDES 2021–2024

**DOI:** 10.1371/journal.pgph.0005891

**Published:** 2026-01-23

**Authors:** Marcelo Cárdenas, Cirene Santana, Alejandra Castro, Antony Gonzales, Guillermo Salvatierra

**Affiliations:** 1 Faculty of Health Sciences, Universidad Peruana de Ciencias Aplicadas, Lima, Peru; 2 Jacobi Medical Center-Albert Einstein College of Medicine, Bronx, New York, United States of America; 3 Faculty of Health Sciences, Universidad Privada del Norte, Lima, Peru; PLOS: Public Library of Science, UNITED STATES OF AMERICA

## Abstract

In Peru, the national immunization schedule includes Bacillus Calmette–Guérin (BCG), Diphtheria–Tetanus–Pertussis (DTaP), Measles–Mumps–Rubella (MMR), and Poliomyelitis (Pol) vaccines. Despite their proven role in reducing infant morbidity and mortality, coverage remains uneven and is potentially influenced by access to communication media. We analyzed data from 37,791 mother–child pairs with children aged 18–24 months from the nationally representative Demographic and Family Health Survey (ENDES) 2021–2024. Using generalized linear models with a Poisson family and log link, we estimated adjusted prevalence ratios (PRa) for associations between complete vaccination and access to various media types (newspaper, radio, television, internet, computer, landline, mobile phone), accounting for the complex survey design through sampling weights, stratification, and clustering. Complete vaccination was reported for 94.1% of children for BCG, 85.5% for DTaP, 86.3% for Pol, and 52.9% for MMR. Mobile phone or smartphone ownership was consistently associated with higher completion across all vaccines, ranging from a 12% increase for BCG (PRa 1.12; 95% CI 1.03–1.21) to a 43% increase for MMR (PRa 1.43; 95% CI 1.15–1.77). Radio access was positively associated with DTaP (PRa 1.03; 95% CI 1.00–1.05) and Pol (PRa 1.04; 95% CI 1.01–1.06). Speaking a language other than Spanish or Quechua was linked to lower coverage, particularly for MMR (PRa 0.67; 95% CI 0.56–0.82) and DTaP (PRa 0.76; 95% CI 0.68–0.86). These findings suggest that expanding culturally and linguistically tailored communication strategies through both traditional and digital media, especially mobile phones and radio, could improve vaccination uptake, particularly for MMR, DTaP, and Pol.

## Introduction

Infectious diseases have historically been a leading cause of death among children, particularly in low- and middle-income countries. Each year, an estimated 10.6 million children under five die, and of the 130 million annual births, approximately 3.8 million die within the first four weeks of life, with three-quarters of these deaths occurring during the first week [1]. Neonates are especially vulnerable due to the immaturity of their immune systems [2]. Vaccination has played a critical role in reducing neonatal and infant mortality, preventing severe illness and long-term disability. Global immunization efforts, led by initiatives such as the Expanded Programme on Immunization and supported by the World Health Organization (WHO) and the United Nations Children’s Fund, have contributed to substantial declines in vaccine-preventable diseases [3].

Peru introduced its national immunization schedule in 1972, including essential vaccines such as Bacillus Calmette–Guérin (BCG), Diphtheria–Tetanus–Pertussis (DTaP), Measles–Mumps–Rubella (MMR), and Poliomyelitis (Pol) [4]. These vaccines target diseases with high morbidity and mortality in early life: BCG reduces severe forms of tuberculosis [5–8]; DTaP prevents conditions such as pertussis, which remains a leading cause of neonatal deaths globally [9]; MMR protects against viral diseases with high fatality in under-immunized populations [10,11]; and Pol has nearly eradicated poliomyelitis worldwide, though vaccine-derived strains remain a challenge [12,13].

Despite the availability of childhood vaccines, disparities in coverage persist, driven by socioeconomic, geographic, and informational barriers. In Peru, complete immunization coverage among children aged 12–23 months dropped to 46.3% in 2020 [14]. Access to media and digital technologies is a key but often overlooked factor in vaccine acceptance in low- and middle-income countries. The Ministry of Health (MINSA) disseminates information on childhood immunization through multiple mass communication channels in Peru. State-owned media outlets, including national television and radio, are commonly used to deliver vaccination-related messages to the general population [15]. In addition, digital platforms and social media are increasingly employed to disseminate health information, potentially expanding the reach of immunization messaging among households with access to these media [16]. Previous studies have shown that media channels can play an important role in shaping beliefs and promoting immunization [17–19], while also facilitating the spread of misinformation that may contribute to vaccine hesitancy when health information is disseminated by non-health professionals [20–22]. In the Peruvian context, one study reported that limited access to media was associated with an 11% reduction in compliance with the childhood vaccination schedule [23]. Nevertheless, evidence from Peru remains limited regarding how access to different types of mass media is associated with childhood vaccination coverage in the post-pandemic period, particularly among children under two years of age, using nationally representative data. This study aimed to examine the association between access to various types of communication media, including newspaper, radio, television, internet, computer, landline, and mobile phone, and completion of the vaccination schedule for BCG, DTaP, MMR, and Pol among children aged 18–24 months in Peru, using nationally representative data from the 2021–2024 Demographic and Family Health Surveys (ENDES).

## Materials and methods

### Ethics statement

This study analyzed de-identified, publicly available ENDES data, ensuring participant anonymity [24]. Participation in ENDES is voluntary, and informed consent is obtained by INEI at the time of data collection [25]. The survey protocol complies with the DHS Institutional Review Board and U.S. Department of Health and Human Services regulations for human subjects protection (45 CFR 46) [26]. The datasets used in this study were accessed on 30 March 2025 from the official INEI website (https://proyectos.inei.gob.pe/endes/). All datasets are fully anonymized, and the authors did not have access to any information that could identify individual participants. Therefore, no additional ethics approval was required for this secondary analysis.

### Study design and data source

We conducted a secondary data analysis of the 2021–2024 editions of the *Encuesta Demográfica y de Salud Familiar* (ENDES), a nationally and regionally representative cross-sectional survey conducted annually by the *Instituto Nacional de Estadística e Informática* (INEI) as part of the Demographic and Health Surveys (DHS) program [27]. ENDES collects detailed information on household conditions, maternal and child health, and vaccination practices through a stratified, multistage, probabilistic sampling design. The survey methodology, including sampling strategy, fieldwork procedures, and data collection instruments, is described in detail in the official technical reports [27].

### Study population

The analysis included women aged 15–49 years with at least one living child aged 18–24 months and complete vaccination information for that child. We excluded records with missing or duplicated information, as well as those not meeting the specified age criteria.

### Outcome variables

The primary outcomes were completion of four vaccines included in Peru’s national immunization schedule: Bacillus Calmette–Guérin (BCG, single dose), Diphtheria–Tetanus–Pertussis (DTaP, three doses), Measles–Mumps–Rubella (MMR, two doses measles component), and Poliomyelitis (Pol, three doses). Each was coded as a binary variable indicating completion according to the child’s age and national guidelines (4). Prevalence estimates reflect vaccination status at the time of the household survey, based on data collected between 2021 and 2024.

### Exposure variables

Main exposures were indicators of media and technology access: reading a newspaper (yes/no), listening to the radio (yes/no), television access (no access, open signal, or cable), internet access at home (yes/no), computer with internet (yes/no), landline phone (yes/no), and mobile phone or smartphone ownership (yes/no). All were reported by the mother and referred to the availability of the device or service in the household at the time of the survey. This measure reflected household access rather than frequency, context, or nature of use, and did not capture actual exposure to vaccination-related information or other health content.

### Covariates

We selected covariates a priori based on theoretical relevance and previous literature: maternal age group (15–19, 20–24, 25–29, 30–34, 35–39, 40–44, 45–49), first language (Spanish, Quechua, other Indigenous or foreign languages), region (Lima, rest of the coast, highlands, jungle), place of residence (urban/rural), highest education level (no education/primary, secondary, higher), marital status (single, married/cohabiting, widowed/divorced/separated), and household wealth index (poor, middle, high). Detailed coding for each variable including category definitions is provided in S1 Text.

### Statistical analysis

Absolute and relative frequencies were used to describe sociodemographic characteristics and vaccination coverage. Differences in coverage across categories were assessed using the Rao–Scott Chi-square test. Crude and adjusted prevalence ratios (PR) for each vaccine were estimated using generalized linear models with a Poisson distribution and log link. Poisson models were selected over binomial models due to non-convergence for some outcomes. Variables with a p-value < 0.20 in the bivariate analysis were included in multivariable models. All adjusted models accounted for the complex survey design through sampling weights, stratification, and clustering, implemented with the svy commands in Stata SE 18.0 (StataCorp, College Station, TX, USA).

## Results

### Sociodemographic characteristics, media access, and vaccination coverage

**Fig 1:**
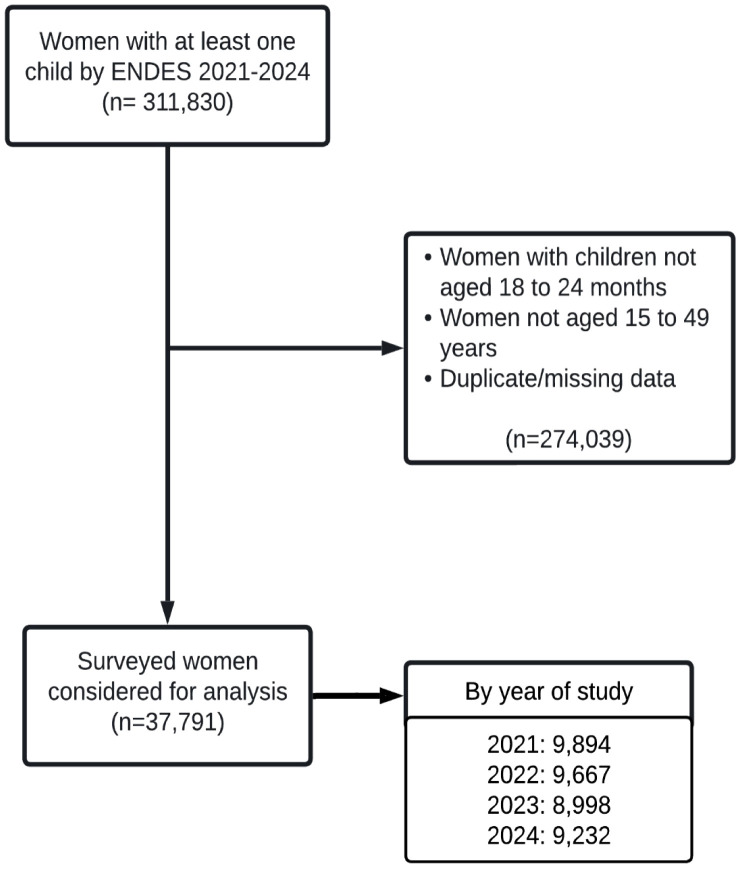
Flowchart for the selection of participants from the Demographic and Family Health Survey (ENDES), Peru, 2021–2024.

The initial dataset included 311,830 participants. After applying exclusion criteria, 274,039 were removed due to missing or duplicated information, children outside the 18–24 month range, or mothers aged under 15 or over 49 years. A total of 37,791 participants were included in the analysis (Fig 1 and S1 Table).

The largest age group was 30–34 years (24.2%), followed by 25–29 years (22.3%) and 35–39 years (19.4%). Most participants reported Spanish as their first language (79.9%), with 15.3% speaking Quechua and 4.8% speaking other Indigenous or foreign languages. By geographic region, 30.1% lived in the rest of the coast, 29.54% in the highlands, 27.9% in the jungle, and 12.5% in Lima. Urban areas accounted for 71.2% of the sample.

Regarding education, 34.1% of participants had higher education, 48.1% had completed secondary school, and 17.6% had primary education or no formal schooling. Most were married or cohabiting (66.1%), while 23.8% were single and 10.1% were widowed, divorced, or separated. Over half of the households (55.2%) were classified as poor, 20.0% as middle wealth, and 24.7% as high wealth.

Access to communication media varied: 30.9% reported reading newspapers, 52.6% listened to the radio, and 78.1% had access to television (47.4% via open signal and 30.7% via cable). Internet access at home was reported by 26.7%, and 22.8% had a computer. While only 5.6% had a landline phone, mobile phone or smartphone ownership was almost universal (96.6%). Vaccination coverage was highest for BCG (94.1%), followed by Pol (86.3%) and DTaP (85.5%). MMR had the lowest coverage, with only 52.9% of children completing the recommended schedule (Table 1).

**Table 1:** Description of sociodemographic variables, communication media, and the vaccine schedule (n = 37,791).

Sociodemographic characteristics	N (%)
**Age group (years)**	
15-19	2,588 (6.9)
20-24	6,293 (16.7)
25-29	8,411 (22.3)
30-34	9,141 (24.2)
35-39	7,335 (19.4)
40-44	3,595 (9.5)
45-49	428 (1.1)
**First-spoken language**	
Spanish	30,197 (79.9)
Quechua	5,782 (15.3)
Others*	1,812 (4.8)
**Region**	
Lima	4,716 (12.5)
Rest of coastline	11,364 (30.1)
Highlands	11,165 (29.5)
Jungle	10,546 (27.9)
**Place of residence**	
Urban	26,920 (71.2)
Rural	10,871 (28.8)
**Highest educational level**	
No education/Elementary	6,633 (17.6)
Secondary	18,281 (48.4)
Higher	12,877 (34.1)
**Marital status (n = 23,196)**	
Single	5,517 (23.8)
Married/ Cohabitant	15,340 (66.1)
Widowed/ Divorced/ Separated	2,339 (10.1)
**Wealth index**	
Poor	20,872 (55.2)
Middle	7,570 (20.0)
High	9,349 (24.7)
**Access to communication media**	**N (%)**
**Newspaper**	
No	26,117 (69.1)
Yes	11,674 (30.9)
**Radio**	
No	17,897 (47.4)
Yes	19,894 (52.6)
**TV**	
No	8,265 (21.9)
Yes, open signal	17,922 (47.4)
Yes, cable	11,604 (30.7)
**Internet access**	
No	27,711 (73.3)
Yes	10,080 (26.7)
**Computer**	
No	29,179 (77.2)
Yes	8,612 (22.8)
**Landline phone**	
No	35,694 (94.5)
Yes	2,097 (5.6)
**Mobile phone/smartphone**	
No	1,307 (3.5)
Yes	36,484 (96.5)
**Vaccine schedule completion**	**N (%)**
**Diphtheria, tetanus, and pertussis (DTaP) vaccine**	
No	5,492 (14.5)
Yes	32,299 (85.5)
**Measles, Mumps, and Rubella (MMR) vaccine**	
No	17,818 (47.2)
Yes	19,973 (52.9)
**Poliomyelitis (Pol) vaccine**	
No	5,189 (13.7)
Yes	32,602 (86.3)
**Bacillus Calmette-Guérin (BCG) vaccine**	
No	2,243 (5.9)
Yes	35,548 (94.1)

*aimara, ashaninka, awajún/aguaruna, shipibo/konibo, shawi/chayahuita, matsigenka/machiguenga, achuar, other native or indigenous languages, portuguese, other foreign language

### DTaP vaccination coverage and associated factors

**Fig 2:**
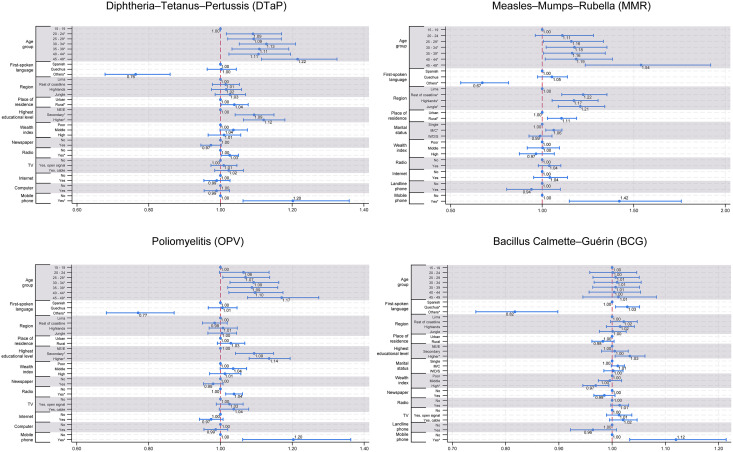
Adjusted prevalence ratios (PRa) with 95% confidence intervals for factors associated with completion of the DTaP, MMR, Pol, and BCG vaccination schedules among children aged 18–24 months, Peru, ENDES 2021–2024. Reference categories: 15–19 years (age group), Spanish (first-spoken language), Lima (region), urban (place of residence), no education/elementary (education level), poor (wealth index), and no access (media variables). Only variables retained in the multivariable models are displayed.

In the multivariable analysis, maternal education, area of residence, radio access, and mobile phone or smartphone ownership were significantly associated with completion of the DTaP vaccination schedule. Speaking a language other than Spanish or Quechua was significantly associated with lower vaccination completion (PRa = 0.76; 95% CI: 0.68-0.86; p < 0.001). Compared to mothers with no formal education, those with higher education had a 12% greater prevalence of completion (PRa = 1.12; 95% CI: 1.06–1.18; p < 0.001). Rural residence was associated with a 4% higher prevalence of complete vaccination (PRa = 1.04; 95% CI: 1.00–1.08; p = 0.042). Listening to the radio was also linked to a 3% higher prevalence (PRa = 1.03; 95% CI: 1.002–1.05; p = 0.034). Finally, owning a mobile phone or smartphone was positively associated with completion (PRa = 1.20; 95% CI: 1.06–1.35; p = 0.003). No significant differences were observed by geographic region after adjustment (Table 2 and Fig 2).

**Table 2:** Adjusted prevalence ratios for determinants of complete vaccination in children aged 18–24 months, Peru, ENDES 2021–2024.

Results	DTaP			MMR			Pol			BCG		
	PRa	95% CI	p	PRa	95% CI	p	PRa	95% CI	p	PRa	95% CI	p
**Sociodemographic Characteristics**												
**Age group (years)**												
15-19	Ref.			Ref.			Ref.			Ref.		
20-24	1.09	1.02 - 1.17	0.016	1.11	0.96 - 1.28	0.152	1.06	1.00 - 1.14	0.060	1.00	0.96 - 1.05	0.958
25-29	1.09	1.02 - 1.17	0.011	1.16	1.01 - 1.33	0.040	1.07	1.01 - 1.14	0.030	1.01	0.96 - 1.05	0.765
30-34	1.13	1.05 - 1.21	0.001	1.18	1.02 - 1.35	0.024	1.09	1.03 - 1.16	0.005	1.01	0.97 - 1.05	0.673
35-39	1.11	1.03 - 1.19	0.005	1.16	1.01 - 1.34	0.039	1.09	1.02 - 1.16	0.010	1.01	0.96 - 1.05	0.768
40-44	1.11	1.02 - 1.20	0.011	1.19	1.02 - 1.38	0.030	1.10	1.02 - 1.17	0.009	1.00	0.96 - 1.05	0.873
45-49	1.22	1.11 - 1.32	<0.001	1.54	1.24 - 1.92	<0.001	1.17	1.08 - 1.27	<0.001	1.01	0.95 - 1.08	0.735
**First-spoken language**												
Spanish	Ref.			Ref.			Ref.			Ref.		
Quechua	1.00	0.96 - 1.04	0.893	1.05	0.97 - 1.14	0.188	1.01	0.97 - 1.05	0.792	1.03	1.01 - 1.05	0.011
Others**	0.76	0.68 - 0.86	<0.001	0.67	0.56 - 0.82	<0.001	0.77	0.68 - 0.87	<0.001	0.82	0.74 - 0.90	<0.001
**Region**												
Lima	Ref.			Ref.			Ref.			Ref.		
Rest of coastline	1.01	0.98 - 1.05	0.444	1.22	1.11 - 1.35	<0.001	0.98	0.95 - 1.02	0.361	1.02	1.00 - 1.05	0.083
Highlands	1.02	0.97 - 1.06	0.450	1.17	1.05 - 1.31	0.003	1.01	0.97 - 1.05	0.726	1.02	0.99 - 1.04	0.255
Jungle	1.03	0.99 - 1.07	0.212	1.21	1.09 - 1.34	<0.001	1.00	0.97 - 1.04	0.840	1.00	0.98 - 1.03	0.940
**Place of residence**												
Urban	Ref.			Ref.			Ref.			Ref.		
Rural	1.04	1.001 - 1.08	0.042	1.11	1.03 - 1.19	0.006	1.03	0.99 - 1.07	0.132	0.98	0.96 - 1.004	0.106
**Highest educational level**												
No education/Elementary	Ref.						Ref.			Ref.		
Secondary	1.09	1.04 - 1.15	<0.001				1.09	1.04 - 1.15	<0.001	1.00	0.98 - 1.03	0.699
Higher	1.12	1.06 - 1.18	<0.001				1.14	1.08 - 1.19	<0.001	1.03	1.01 - 1.06	0.018
**Marital status (n = 23,196)**												
Single				Ref.						Ref.		
Married/ Cohabitant				1.06	1.02 - 1.11	0.004				1.01	1.00 - 1.02	0.089
Widower/ Divorced/ Separated				0.99	0.93 - 1.05	0.727				1.00	0.98 - 1.02	0.799
**Wealth index**												
Poor	Ref.			Ref.			Ref.			Ref.		
Middle	1.04	1.00 - 1.07	0.058	1.00	0.92 - 1.09	0.995	1.04	1.00 - 1.07	0.055	1.00	0.97 - 1.02	0.704
High	1.01	0.97 - 1.06	0.662	0.97	0.88 - 1.07	0.493	1.01	0.97 - 1.06	0.570	0.97	0.95 - 0.99	0.015
**Access to communication media**												
**Newspaper**												
No	Ref.						Ref.			Ref.		
Yes	0.97	0.95 - 1.00	0.071				0.98	0.95 - 1.01	0.145	0.99	0.97 - 1.01	0.152
**Radio**												
No	Ref.			Ref.			Ref.			Ref.		
Yes	1.03	1.002 - 1.05	0.034	1.04	0.98 - 1.10	0.207	1.04	1.01 - 1.06	0.001	1.01	1.00 - 1.03	0.095
**TV**												
No	Ref.						Ref.			Ref.		
Yes, open signal	1.01	0.97 - 1.04	0.624				1.03	0.99 - 1.06	0.183	1.01	0.99 - 1.04	0.278
Yes, cable	1.02	0.98 - 1.06	0.251				1.04	1.00 - 1.08	0.720	1.02	1.00 - 1.05	0.113
**Internet access**												
No	Ref.			Ref.			Ref.					
Yes	0.99	0.95 - 1.03	0.577	1.04	0.95 - 1.14	0.365	0.97	0.94 - 1.01	0.125			
**Computer**												
No	Ref.						Ref					
Yes	0.99	0.95 - 1.03	0.550				0.99	0.95 - 1.02	0.435			
**Landline phone**												
No				Ref.						Ref.		
Yes				0.94	0.81 - 1.10	0.445				0.96	0.92 - 1.01	0.109
**Mobile phone/smartphone**												
No	Ref.			Ref.			Ref.			Ref.		
Yes	1.20	1.06 - 1.35	0.003	1.43	1.15 - 1.77	0.001	1.20	1.06 - 1.36	0.004	1.12	1.03 - 1.21	0.006

PRa: Adjusted prevalence ratio.

**aimara, ashaninka, awajún/aguaruna, shipibo/konibo, shawi/chayahuita, matsigenka/machiguenga, achuar, other native or indigenous languages, portuguese, other foreign language. Blank: Variables excluded in the multivariable regression model

Across the years, several regions consistently reached very high levels of DTaP coverage. In 2021, Cajamarca (97.04%, 280/285), Pasco (90.2%, 275/305) and Huancavelica (89.8%, 237/264) showed the highest proportions of completed schedules. In 2022, Junin (93.9%, 355/378), Moquegua (92.5%, 284/307) and Ancash (90.1%, 319/354). In 2023, Tumbes (94.2%, 386/410), Ancash (93.3%, 279/299) and Cusco (93.1%, 203/218) had the highest coverage. While in 2024, Tumbes (94.4%, 352/373), Moquegua (93.8%, 273/291) and Piura (93.0%, 359/386) were the ones with the highest prevalence. In all four years, the majority of vaccinated mothers resided in urban areas (83.5%, 85.2%, 87.5%, and 89.8%, respectively). These results reinforce the pattern observed in the national estimates, confirming DTaP as the vaccine with the highest and most stable coverage across regions and years (S1 and S2 Fig). Detailed bivariate analyses for DTaP vaccine are provided in S2 and S6 Tables.

### MMR vaccination coverage and associated factors

Only 52.85% of children completed the full MMR vaccination schedule, making it the lowest among all vaccines evaluated. In the multivariable regression, speaking a language other than Spanish or Quechua was associated with 33% lower coverage (PRa = 0.67; 95% CI: 0.56–0.82; p < 0.001). Compared to mothers living in Lima, those in the rest of the coast (PRa = 1.22; 95% CI: 1.11–1.35; p < 0.001), highlands (PRa = 1.17; 95% CI: 1.05–1.31; p = 0.003), and jungle (PRa = 1.21; 95% CI: 1.09–1.34; p < 0.001) had significantly higher coverage. Rural residence remained positively associated (PRa = 1.11; 95% CI: 1.03–1.19; p = 0.006). Among media-related variables, owning a mobile phone or smartphone was linked to 43% higher coverage compared to those without one (PRa = 1.43; 95% CI: 1.15–1.77; p = 0.001) (Table 2 and Fig 2).

At the regional level, in 2021, the highest MMR coverage was observed in Cajamarca (65.5%, 199/304), Ayacucho (62.2%, 220/354) and Pasco (59.7%, 182/305), with 47.6% of mothers living in urban areas (3331/6994). In 2022, leading regions were Áncash (68.1%, 241/354), La Libertad (66.4%, 237/357) and Moquegua (62.5%, 192/307), with 47.7% urban mothers (3282/6884). In 2023, Cusco (69.7%, 152/218), Huánuco (68.3%, 230/337) and La Libertad (67.7%, 239/353) led, with 51.7% of mothers residing in urban areas (3357/6494). Piura (77.7%, 300/386), Áncash (76.5%, 163/213) and La Libertad (72.5%, 251/346) had the highest vaccination coverage in 2024. These trends show progress in specific regions despite overall low national coverage (S3 and S4 Fig.). Detailed bivariate analyses for MMR vaccine are provided in S3 and S7 Tables.

### Poliomyelitis vaccination coverage and associated factors

In the multivariable analysis, several factors were significantly associated with Pol completion. Mothers aged 25–29 years (PRa = 1.07; 95% CI: 1.01-1.14; p = 0.03), 30–34 years (PRa = 1.09; 95% CI: 1.03-1.16; p = 0.005), 35–39 years (PRa = 1.09; 95% CI: 1.02-1.16; p = 0.01), 40–44 years (PRa = 1.1; 95% CI: 1.02-1.17; p = 0.009), and 45–49 years (PRa = 1.17; 95% CI: 1.08-1.27; p < 0.001) had higher adjusted prevalence ratios compared to those aged 15–19 years. Speaking a language other than Spanish or Quechua was associated with a lower prevalence of full vaccination (PRa = 0.77; 95% CI: 0.68-0.87; p < 0.001). Higher (PRa = 1.14; 95% CI: 1.08-1.19; p < 0.001) and secondary (PRa = 1.09; 95% CI: 1.04-1.15; p < 0.001) education were positively associated with coverage. Among media and technology variables, listening to the radio (PRa = 1.04; 95% CI: 1.01-1.06; p = 0.001) and owning a mobile phone or smartphone (PRa = 1.2; 95% CI: 1.06-1.36; p = 0.004) remained independently associated with higher coverage (Table 2 and Fig 2).

At the regional level, the highest Pol coverage in 2021 was observed in Cajamarca (94.4%, 287/304), Apurímac (93.2%, 303/325), and Pasco (90.5%, 276/305). In 2022, La Libertad (93.00%, 332/357), Junín (92.3%, 349/378), and Callao (90.9%, 359/395) ranked highest. In 2023, the leading regions were San Martín (96.8%, 368/380), Apurímac (96.3%, 282/293), and Arequipa (95.0%, 282/297). In 2024, Tumbes (95.4%, 356/373), Moquegua (94.5%, 275/291), and Huánuco (94.1%, 412/438) achieved the highest prevalence. Across all four years, the majority of vaccinated mothers resided in urban areas, representing 85.0% (5948/6994) in 2021, 85.9% (5913/6884) in 2022, 88.7% (5758/6494) in 2023, and 90.1% (5898/6548) in 2024 (S5 and S6 Fig). Detailed bivariate analyses for Pol vaccine are provided in S4 and S8 Tables.

### BCG vaccination coverage and associated factors

In the multivariable analysis, several variables remained significantly associated with BCG completion. Compared to mothers who spoke Spanish, those who spoke Quechua had a 3% higher prevalence (PRa = 1.03; 95% CI: 1.01-1.05; p = 0.011), while those speaking other Indigenous or foreign languages had an 18% lower prevalence (PRa = 0.82; 95% CI: 0.74–0.90; p < 0.001). Mothers with higher education showed a 3% greater prevalence of full BCG vaccination compared to those without formal education (PRa = 1.03; 95% CI: 1.01–1.06; p = 0.018). Belonging to the high wealth index group was associated with a slightly lower coverage (PRa = 0.97; 95% CI: 0.95–0.99; p = 0.015). Mobile phone or smartphone ownership was strongly associated with coverage, with a 12% higher prevalence among owners (PRa = 1.12; 95% CI: 1.03–1.21; p = 0.006) (Table 2 and Fig 2).

At the regional level, in 2021, the highest coverage rates were observed in Piura (98.8%, 421/426), Callao (97.2%, 414/426), and Cajamarca (97.0%, 295/304), with 83.5% (5843/6994) of mothers residing in urban areas. In 2022, the highest coverage was reported in Huancavelica (99.1%, 313/316), Huánuco (98.9%, 359/363), and Tacna (98.8%, 328/332), with 85.2% (5866/6884) of mothers living in urban areas. In 2023, Apurímac, Arequipa, Huancavelica, Madre de Dios, and Piura achieved 100.0% BCG vaccination coverage, with 87.5% (5679/6494) of vaccinated children residing in urban areas. Lastly, in 2024 Apurímac and Callao reached a 100.00% coverage, followed by Piura with 99.2% (383/386), and a percentage of 89.% (5879/6548) of mothers being from urban areas (S7 and S8 Fig). Detailed bivariate analyses for BCG vaccine are provided in S6 and S9 Tables.

## Discussion

This study highlights persistent gaps in vaccination coverage among Peruvian children under two years of age between 2021 and 2024. While BCG vaccination maintained high coverage rates consistent with WHO targets [28], DTaP, Pol, and particularly MMR exhibited suboptimal completion [29,30]. The contrast between BCG (94.06%) and MMR (52.85%) coverage is particularly concerning, given the increased risk of measles outbreaks when coverage falls below the 95% threshold. DTaP and Pol coverage, although higher than MMR, still remained below the 90% benchmark, indicating systemic vulnerabilities in the national immunization program. In contrast to other vaccines, BCG is administered immediately after birth at the health center and requires only one dose, which likely contributes to its consistently high coverage [4]. Conversely, the lower completion of MMR may be explained by its longer dosing schedule, with a six-month interval between doses [4]. In addition, MMR uptake was adversely affected by disruptions to routine immunization services during the COVID-19 pandemic, which led to substantial declines in coverage across the Americas, reaching 81% for the first dose and 70% for the second dose [31]. Together, these factors likely hindered caregivers’ ability to complete the full MMR immunization schedule.

Across all vaccines, mobile phone or smartphone ownership was consistently associated with higher completion, with adjusted prevalence ratios indicating increases from 15% (Pol) to 34% (MMR) compared with mothers without such devices. These results align with international evidence showing the effectiveness of mobile-based interventions, such as SMS reminders, voice calls, and health applications, in improving immunization timeliness and adherence [32–35]. In contexts with fragmented health service access, mobile phones can help bridge information gaps, provide timely reminders, and support decision-making [36]. However, as ENDES only measures ownership and not usage patterns, it remains unknown whether these devices were used to access health information, receive reminders, or engage with social media. The association observed could also reflect reverse causation, where mothers already more engaged with healthcare are also more likely to own a mobile phone. Future research should investigate usage patterns, as access alone does not ensure effective or accurate information flow. Furthermore, given the documented spread of vaccine misinformation through these same platforms, interventions should be coupled with robust, culturally relevant health literacy strategies [37,38].

Language barriers emerged as another structural determinant of vaccination inequities. Mothers speaking a language other than Spanish or Quechua consistently showed lower vaccine completion, with differences ranging from 14% (DTaP, Pol) to 34% (MMR). This finding underscores longstanding inequities in health communication and service accessibility for linguistically marginalized populations, that reported in previous study [39]. In multilingual settings such as Peru, where more than 40 Indigenous languages are spoken [40], a lack of linguistically tailored health messaging may hinder comprehension of vaccination schedules and reduce trust in services. Digital platforms such as TikTok and Instagram show the Ministry of Health’s official accounts promoting key neonatal vaccines; however, most content is in Spanish, indicating a lack of linguistic correspondence given the country’s diversity, and there is currently no formal evidence available to assess the population-level impact of these initiatives. Language mismatch can limit understanding, weaken engagement with health services, and ultimately decrease vaccine uptake [41,42]. Addressing this challenge requires culturally grounded communication strategies, trained community health workers fluent in local languages, and the intentional inclusion of linguistic diversity in national immunization planning.

Geographic patterns revealed important disparities. Although urban areas concentrated more vaccinated children in absolute terms, proportional coverage did not always favor them. MMR coverage, for example, was higher in rural than in urban areas, a contradictory finding given the presumed advantage of urban settings in service accessibility. Possible explanations include targeted outreach interventions in rural communities, such as periodic vaccination campaigns and more consistent contact with community health workers, who often play a central role in immunization adherence. In recent years, the Peruvian government has also implemented programs to strengthen multilingual health communication and expand healthcare infrastructure in rural regions [43–45], which may have contributed to these trends. MMR also showed higher rates in the rest of the coastline, highlands, and jungle regions. Despite targeted efforts such as population education campaigns and the use of solar-powered refrigerators to maintain cold chains in remote communities [46–48], barriers including long distances to health facilities, lack of electricity, and maternal hesitancy still hinder timely completion of vaccination schedules [49,50]. Another unexpected finding was the slightly lower BCG coverage among households in the highest wealth index group, which could reflect differences in delivery settings: wealthier families may rely more on private clinics where BCG administration is less systematically recorded in national registries or the use of alternative vaccination schedules. These patterns have not yet been sufficient to reach the WHO-recommended coverage of over 90% needed to control the spread of vaccine-preventable diseases [51] and warrant further investigation through qualitative approaches and health service delivery audits.

Access to mass media also showed a modest positive association with vaccination, though the magnitude of association was small. Radio access remained statistically significant for DTaP and Pol even after adjustment, with increases of around 3–4%. While this suggests that radio continues to play a role in areas with limited access to other media, its association size is limited, and the potential for impact may be greater when integrated into multifaceted communication strategies. Digital platforms, often accessed through mobile phones, present a dual challenge: they can facilitate rapid dissemination of accurate health information but can also amplify unverified or false content [37,38,52]. As ENDES does not currently measure exposure to health content or misinformation, future surveys could incorporate such variables to better understand the digital information landscape.

This study has several limitations. First, the cross-sectional nature of the ENDES data prevents establishing causal relationships between exposure variables and vaccination completion, and reverse causation remains a possibility. Second, vaccination status was based on maternal self-report, which is susceptible to recall bias, particularly for vaccines administered long before the interview. Third, while ENDES is nationally representative, it may not capture hyperlocal or community-specific barriers, especially in remote or Indigenous areas. Although ENDES does not collect direct measures of physical accessibility such as distance or travel time to health facilities, the inclusion of area of residence, region, and household wealth index partially captures structural and geographical differences in access to vaccination services. Fourth, despite adjusting for multiple sociodemographic variables, residual confounding from unmeasured factors such as healthcare accessibility, maternal beliefs, or health literacy cannot be ruled out. Fifth, the ENDES survey does not include data on the ownership of internet-enabled devices other than smartphones and computers, which prevents us from measuring the influence of other devices Finally, the measurement of media access was limited to the reported availability of devices or services in the household, without assessing actual use, frequency, or the type of content consumed. These aspects are critical to determine whether access translates into meaningful exposure to vaccination-related information or other health content. Consequently, observed associations may reflect indirect socioeconomic influences, behavioral patterns, or other contextual factors rather than direct associations of information exposure. Future research should incorporate measures of usage patterns, content type, and information quality to better elucidate the mechanisms linking media access and vaccination uptake.

Overall, these findings reinforce that vaccination coverage is shaped not only by the availability of vaccines but also by broader social, cultural, and technological determinants. Language barriers, unequal access to communication technologies, and geographic disparities remain critical challenges to equitable immunization in Peru. Addressing these will require coordinated, multisectoral strategies that move beyond service provision to integrate culturally and linguistically appropriate communication, targeted outreach to underserved regions, and the safe and effective use of digital tools. Such approaches are essential to ensure that all children in Peru, and in similar settings, receive complete and timely vaccination as both a public health necessity and an equity imperative.

## Supporting information

S1 Text ENDES’ questions used as variables in the study. (DOCX)

S1 Table Flow of study participants and reasons for exclusion, ENDES 2021–2024. (XLSX)

S2 Table Sociodemographic and social media factors associated with completing the Diphtheria, Tetanus and Pertussis (DTaP) vaccination schedule. (XLSX)

S3 Table Sociodemographic and media factors associated with completing the Measles/Mumps/Rubella (MMR) vaccination schedule. (XLSX)

S4 Table Sociodemographic and media factors associated with completing the Poliomyelitis (Pol) vaccination schedule. (XLSX)

S5 Table Sociodemographic and media factors associated with completing the Bacillus Calmette-Guérin (BCG) vaccination schedule. (XLSX)

S6 Table Crude and prevalence ratios for determinants of complete vaccination for DTaP. (XLSX)

S7 Table Crude and prevalence ratios for determinants of complete vaccination for MMR. (XLSX)

S8 Table Crude and prevalence ratios for determinants of complete vaccination for Pol. (XLSX)

S9 Table Crude and prevalence ratios for determinants of complete vaccination for BCG. (XLSX)

S1 Fig Frequency of mothers who completed DTaP vaccination schedule through the years 2021 and 2022. A1: Percentage by region in 2021. B1: Percentage by place of residence in 2021. A2: Percentage by region in 2022. B2: Percentage by place of residence in 2022. Departmental boundaries obtained from official shapefiles of the Instituto Nacional de Estadística e Informática (INEI), accessed via GeoGPS Perú (https://www.geogpsperu.com/2019/08/limite-departamental-politico-shapefile.html). Licensed under the Open Data Commons Attribution License (https://datosabiertos.gob.pe/dataset/limites-departamentales), compatible with CC BY 4.0.
(TIF)

S2 Fig Frequency of mothers who completed DTaP vaccination schedule through the years 2023 and 2024. A1: Percentage by region in 2023. B1: Percentage by place of residence in 2023. A2: Percentage by region in 2024. B2: Percentage by place of residence in 2024. Departmental boundaries obtained from official shapefiles of the Instituto Nacional de Estadística e Informática (INEI), accessed via GeoGPS Perú (https://www.geogpsperu.com/2019/08/limite-departamental-politico-shapefile.html). Licensed under the Open Data Commons Attribution License (https://datosabiertos.gob.pe/dataset/limites-departamentales), compatible with CC BY 4.0.
(TIF)

S3 Fig Frequency of mothers who completed MMR vaccination schedule through the years 2021 and 2022. A1: Percentage by region in 2021. B1: Percentage by place of residence in 2021. A2: Percentage by region in 2022. B2: Percentage by place of residence in 2022. Departmental boundaries obtained from official shapefiles of the Instituto Nacional de Estadística e Informática (INEI), accessed via GeoGPS Perú (https://www.geogpsperu.com/2019/08/limite-departamental-politico-shapefile.html). Licensed under the Open Data Commons Attribution License (https://datosabiertos.gob.pe/dataset/limites-departamentales), compatible with CC BY 4.0.
(TIF)

S4 Fig Frequency of mothers who completed MMR vaccination schedule through the years 2023 and 2024. A1: Percentage by region in 2023. B1: Percentage by place of residence in 2023. A2: Percentage by region in 2024. B2: Percentage by place of residence in 2024. Departmental boundaries obtained from official shapefiles of the Instituto Nacional de Estadística e Informática (INEI), accessed via GeoGPS Perú (https://www.geogpsperu.com/2019/08/limite-departamental-politico-shapefile.html). Licensed under the Open Data Commons Attribution License (https://datosabiertos.gob.pe/dataset/limites-departamentales), compatible with CC BY 4.0.
(TIF)

S5 Fig Frequency of mothers who completed Pol vaccination schedule through the years 2021 and 2022. A1: Percentage by region in 2021. B1: Percentage by place of residence in 2021. A2: Percentage by region in 2022. B2: Percentage by place of residence in 2022. Departmental boundaries obtained from official shapefiles of the Instituto Nacional de Estadística e Informática (INEI), accessed via GeoGPS Perú (https://www.geogpsperu.com/2019/08/limite-departamental-politico-shapefile.html). Licensed under the Open Data Commons Attribution License (https://datosabiertos.gob.pe/dataset/limites-departamentales), compatible with CC BY 4.0.
(TIF)

S6 Fig Frequency of mothers who completed Pol vaccination schedule through the years 2023 and 2024. A1: Percentage by region in 2023. B1: Percentage by place of residence in 2023. A2: Percentage by region in 2024. B2: Percentage by place of residence in 2024. Departmental boundaries obtained from official shapefiles of the Instituto Nacional de Estadística e Informática (INEI), accessed via GeoGPS Perú (https://www.geogpsperu.com/2019/08/limite-departamental-politico-shapefile.html). Licensed under the Open Data Commons Attribution License (https://datosabiertos.gob.pe/dataset/limites-departamentales), compatible with CC BY 4.0.
(TIF)

S7 Fig Frequency of mothers who completed BCG vaccination schedule through the years 2021 and 2022. A1: Percentage by region in 2021. B1: Percentage by place of residence in 2021. A2: Percentage by region in 2022. B2: Percentage by place of residence in 2022. Departmental boundaries obtained from official shapefiles of the Instituto Nacional de Estadística e Informática (INEI), accessed via GeoGPS Perú (https://www.geogpsperu.com/2019/08/limite-departamental-politico-shapefile.html). Licensed under the Open Data Commons Attribution License (https://datosabiertos.gob.pe/dataset/limites-departamentales), compatible with CC BY 4.0.
(TIF)

S8 Fig Frequency of mothers who completed BCG vaccination schedule through the years 2023 and 2024. A1: Percentage by region in 2023. B1: Percentage by place of residence in 2023. A2: Percentage by region in 2024. B2: Percentage by place of residence in 2024. Departmental boundaries obtained from official shapefiles of the Instituto Nacional de Estadística e Informática (INEI), accessed via GeoGPS Perú (https://www.geogpsperu.com/2019/08/limite-departamental-politico-shapefile.html). Licensed under the Open Data Commons Attribution License (https://datosabiertos.gob.pe/dataset/limites-departamentales), compatible with CC BY 4.0.
(TIF)
