# Supplementary material for: The role of the media in the coverage of childhood vaccination in children under two years of age in Peru, ENDES 2021–2024
Source: PLOS Glob Public Health. 2026 Jan 23;6(1):e0005891. doi: 10.1371/journal.pgph.0005891 (PMC12829941; doi:10.1371/journal.pgph.0005891)
Supplement: S1 Text — (DOCX) [file pgph.0005891.s001.docx]

| **S1 Text. ENDES’ questions used as variables in the study.** | | | | |
| --- | --- | --- | --- | --- |
| **Variable name (English)** | **Question (Spanish)** | **Code** | **Alternatives (Spanish)** | |
| Age group (years) | Años cumplidos | QS23 | - | - |
| First-spoken language | Cuál es el idioma o lengua materna que aprendió hablar en su niñez | QS25AA | 1. | Quechua |
|  |  |  | 2. | Aymara |
|  |  |  | 3. | Ashaninka |
|  |  |  | 4. | Awajn/Aguaruna |
|  |  |  | 5. | Shipibo/Conibo |
|  |  |  | 6. | Shawi/Chayahuita |
|  |  |  | 7. | Matsigenka/Machiguenga |
|  |  |  | 8. | Achuar |
|  |  |  | 9. | Otra lengua nativa u originaria |
|  |  |  | 10. | Castellano |
|  |  |  | 11. | Portugués |
|  |  |  | 12. | Otra lengua extranjera |
| Region | Región | HV024 | 1. | Amazonas |
|  |  |  | 2. | Ancash |
|  |  |  | 3. | Apurímac |
|  |  |  | 4. | Arequipa |
|  |  |  | 5. | Ayacucho |
|  |  |  | 6. | Cajamarca |
|  |  |  | 7. | Callao |
|  |  |  | 8. | Cusco |
|  |  |  | 9. | Huancavelica |
|  |  |  | 10. | Huánuco |
|  |  |  | 11. | Ica |
|  |  |  | 12. | Junín |
|  |  |  | 13. | La Libertad |
|  |  |  | 14. | Lambayeque |
|  |  |  | 15. | Lima |
|  |  |  | 16. | Loreto |
|  |  |  | 17. | Madre de Dios |
|  |  |  | 18. | Moquegua |
|  |  |  | 19. | Pasco |
|  |  |  | 20. | Piura |
|  |  |  | 21. | Puno |
|  |  |  | 22. | San Martín |
|  |  |  | 23. | Tacna |
|  |  |  | 24. | Tumbes |
|  |  |  | 25. | Ucayali |
| Place of residence | Área de residencia | HV025 | 1. | Área urbana |
|  |  |  | 2. | Área rural |
| Highest educational level | Nivel que aprobó | QS25N | 0. | Inicial / pre-escolar |
|  |  |  | 1. | Primaria |
|  |  |  | 2. | Secundaria |
|  |  |  | 3. | Superior no universitario |
|  |  |  | 4. | Superior universitario |
|  |  |  | 5. | Postgrado |
| Marital status | Cuál es su estado civil o conyugal | HV115 | 1. | Conviviente |
|  |  |  | 2. | Casado(a) |
|  |  |  | 3. | Viudo(a) |
|  |  |  | 4. | Divorciado(a) |
|  |  |  | 5. | Separado(a) |
|  |  |  | 6. | Soltero(a) |
| Wealth index | Índice de riqueza | HV270 | 1. | Lo más pobre |
|  |  |  | 2. | Pobre |
|  |  |  | 3. | Medio |
|  |  |  | 4. | Rico |
|  |  |  | 5. | Más Rico |
| Newspaper | Frecuencia de lectura de un periódico o revista | V157 | 1. | Casi todos los días |
|  |  |  | 2. | Una vez por semana |
|  |  |  | 3. | De vez en cuando |
|  |  |  | 4. | Nunca |
| Radio | En su hogar tiene: radio | V120 | 1. | Sí |
|  |  |  | 2. | No |
| TV | En su hogar tiene: televisión | HV208 | 1. | Sí |
|  |  |  | 2. | No |
| Internet access | Tiene acceso a Internet en casa | SH61Q | 1. | Sí |
|  |  |  | 2. | No |
| Computer | Tiene computadora | SH61P | 1. | Sí |
|  |  |  | 2. | No |
| Landline phone | En su hogar tiene: teléfono fijo | HV221 | 1. | Sí |
|  |  |  | 2. | No |
| Mobile phone/smartphone | Tiene un teléfono móvil (celular) | HV243A | 1. | Sí |
|  |  |  | 2. | No |
| Diphtheria, tetanus, and pertussis (DTaP) vaccine | Vacuna DPT | H7 | 1. | Al final de la entrevista copie del carné las fechas de vacunación para cada vacuna |
|  |  |  | 2. | Anote "44" en la columna da si la tarjeta muestra que se dio una vacuna pero que no se anote la fecha |
| Measles, Mumps, and Rubella (MMR) vaccine | Recibí vacuna contra el SARAMPIÓN | H9 | 1. | Al final de la entrevista copie del carné las fechas de vacunación para cada vacuna |
|  |  |  | 2. | Anote "44" en la columna da si la tarjeta muestra que se dio una vacuna pero que no se anote la fecha |
| Poliomyelitis (Pol) vaccine | Recibió Vacuna POLIO | H8 | 1. | Al final de la entrevista copie del carné las fechas de vacunación para cada vacuna |
|  |  |  | 2. | Anote "44" en la columna da si la tarjeta muestra que se dio una vacuna pero que no se anote la fecha |
| Bacillus Calmette-Guérin (BCG) vaccine | Recibí vacuna BCG | H2 | 1. | Al final de la entrevista copie del carné las fechas de vacunación para cada vacuna |
|  |  |  | 2. | Anote "44" en la columna da si la tarjeta muestra que se dio una vacuna pero que no se anote la fecha |

<http://webinei.inei.gob.pe/anda_inei/index.php/catalog/760/vargrp/VG2>
