# Supplementary figures and images for: The role of the media in the coverage of childhood vaccination in children under two years of age in Peru, ENDES 2021–2024
Source: PLOS Glob Public Health. 2026 Jan 23;6(1):e0005891. doi: 10.1371/journal.pgph.0005891 (PMC12829941; doi:10.1371/journal.pgph.0005891)

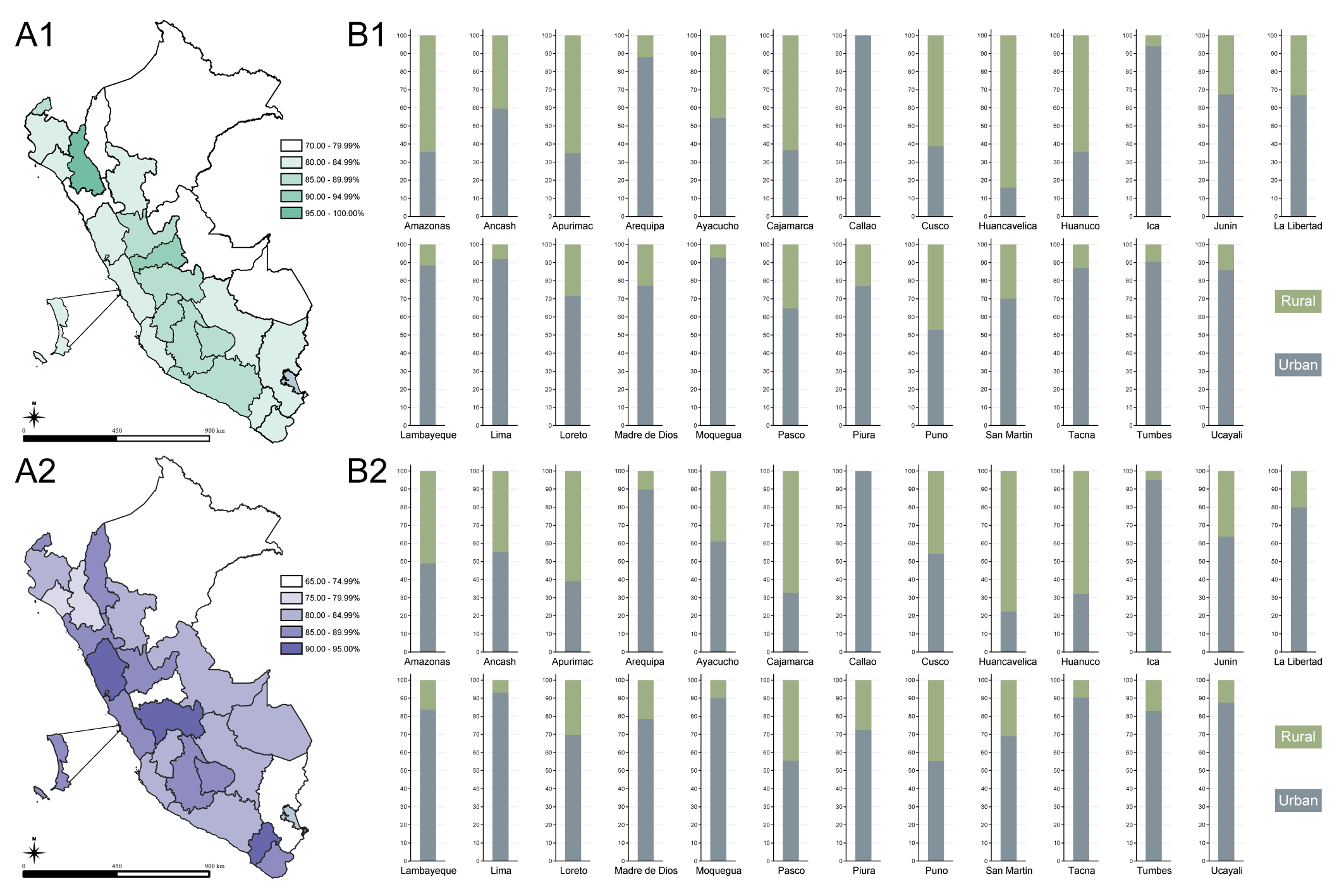

Supplement: S1 Fig — A1: Percentage by region in 2021. B1: Percentage by place of residence in 2021. A2: Percentage by region in 2022. B2: Percentage by place of residence in 2022. Departmental boundaries obtained from official shapefiles of the Instituto Nacional de Estadística e Informática (INEI), accessed via GeoGPS Perú (https://www.geogpsperu.com/2019/08/limite-departamental-politico-shapefile.html). Licensed under the Open Data Commons Attribution License (https://datosabiertos.gob.pe/dataset/limites-departamentales), compatible with CC BY 4.0. (TIF) [file pgph.0005891.s011.tif]

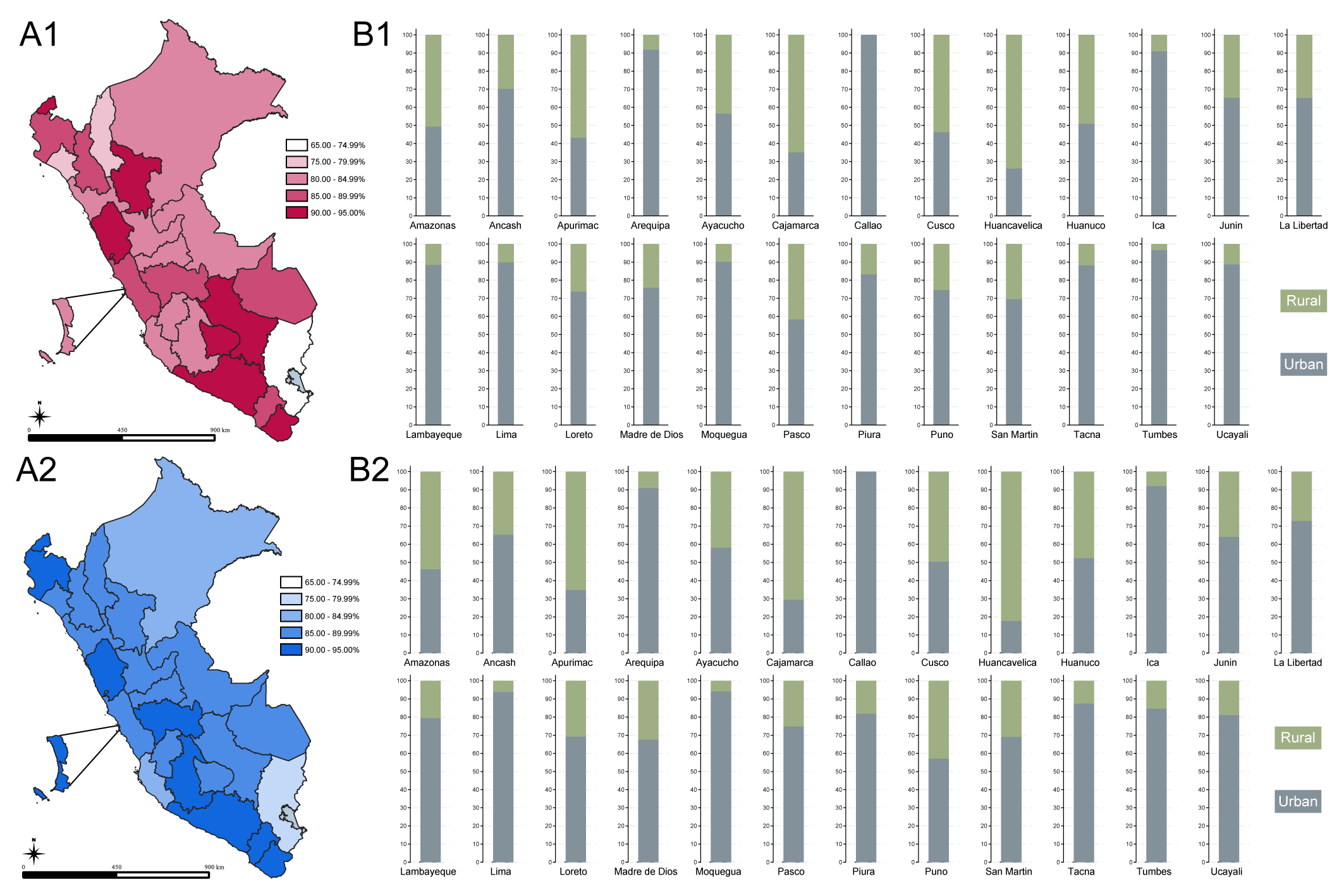

Supplement: S2 Fig — A1: Percentage by region in 2023. B1: Percentage by place of residence in 2023. A2: Percentage by region in 2024. B2: Percentage by place of residence in 2024. Departmental boundaries obtained from official shapefiles of the Instituto Nacional de Estadística e Informática (INEI), accessed via GeoGPS Perú (https://www.geogpsperu.com/2019/08/limite-departamental-politico-shapefile.html). Licensed under the Open Data Commons Attribution License (https://datosabiertos.gob.pe/dataset/limites-departamentales), compatible with CC BY 4.0. (TIF) [file pgph.0005891.s012.tif]

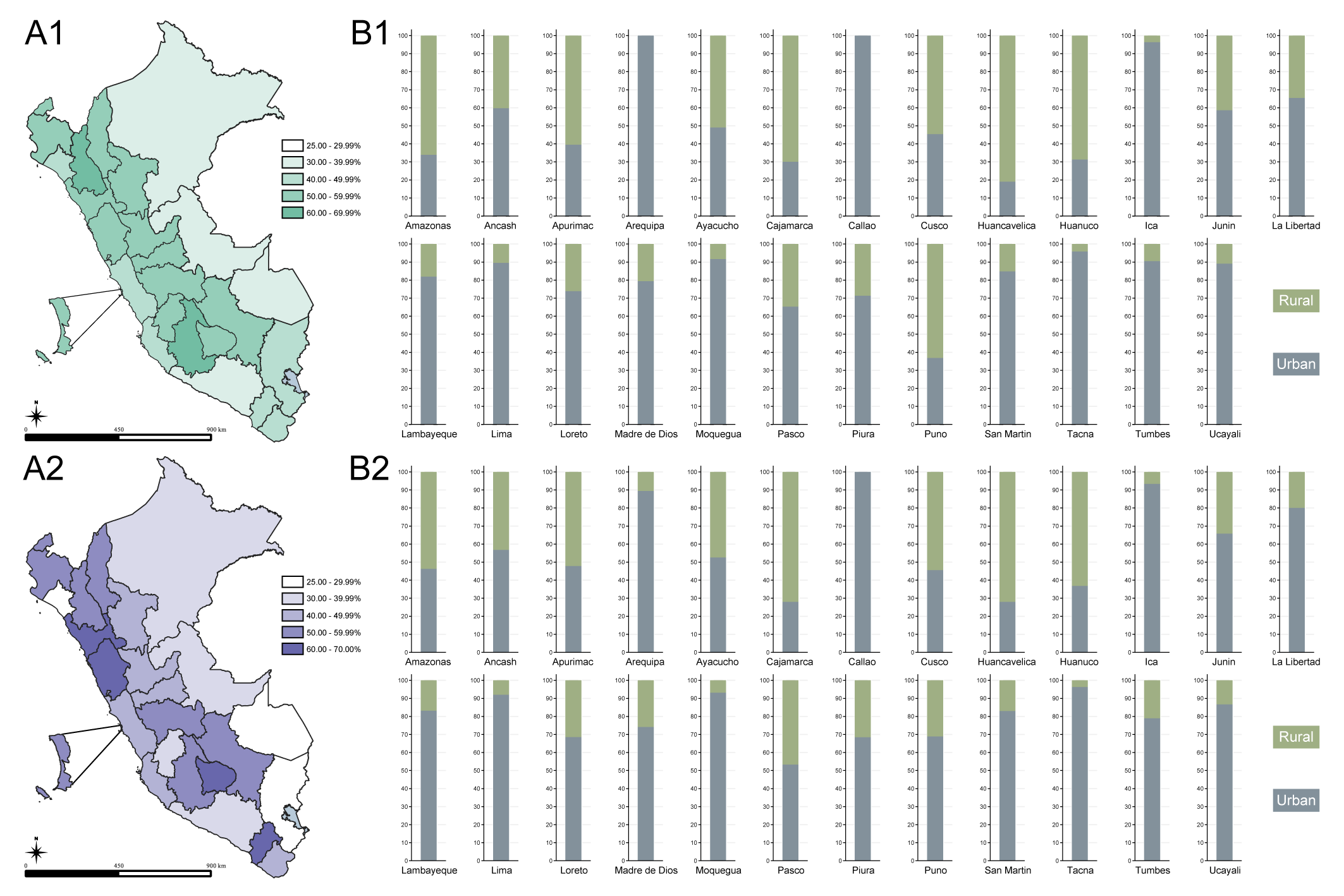

Supplement: S3 Fig — A1: Percentage by region in 2021. B1: Percentage by place of residence in 2021. A2: Percentage by region in 2022. B2: Percentage by place of residence in 2022. Departmental boundaries obtained from official shapefiles of the Instituto Nacional de Estadística e Informática (INEI), accessed via GeoGPS Perú (https://www.geogpsperu.com/2019/08/limite-departamental-politico-shapefile.html). Licensed under the Open Data Commons Attribution License (https://datosabiertos.gob.pe/dataset/limites-departamentales), compatible with CC BY 4.0. (TIF) [file pgph.0005891.s013.tif]

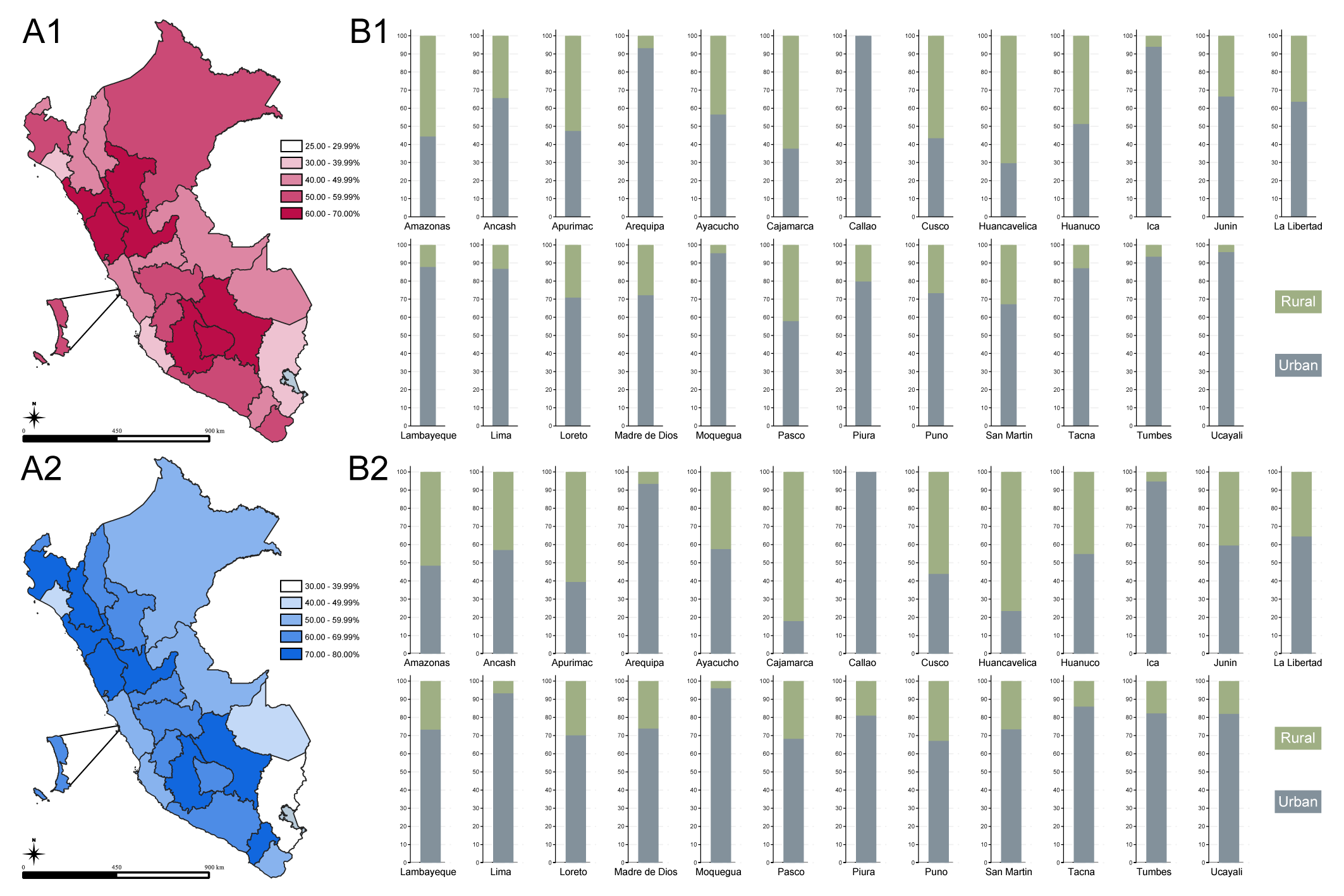

Supplement: S4 Fig — A1: Percentage by region in 2023. B1: Percentage by place of residence in 2023. A2: Percentage by region in 2024. B2: Percentage by place of residence in 2024. Departmental boundaries obtained from official shapefiles of the Instituto Nacional de Estadística e Informática (INEI), accessed via GeoGPS Perú (https://www.geogpsperu.com/2019/08/limite-departamental-politico-shapefile.html). Licensed under the Open Data Commons Attribution License (https://datosabiertos.gob.pe/dataset/limites-departamentales), compatible with CC BY 4.0. (TIF) [file pgph.0005891.s014.tif]

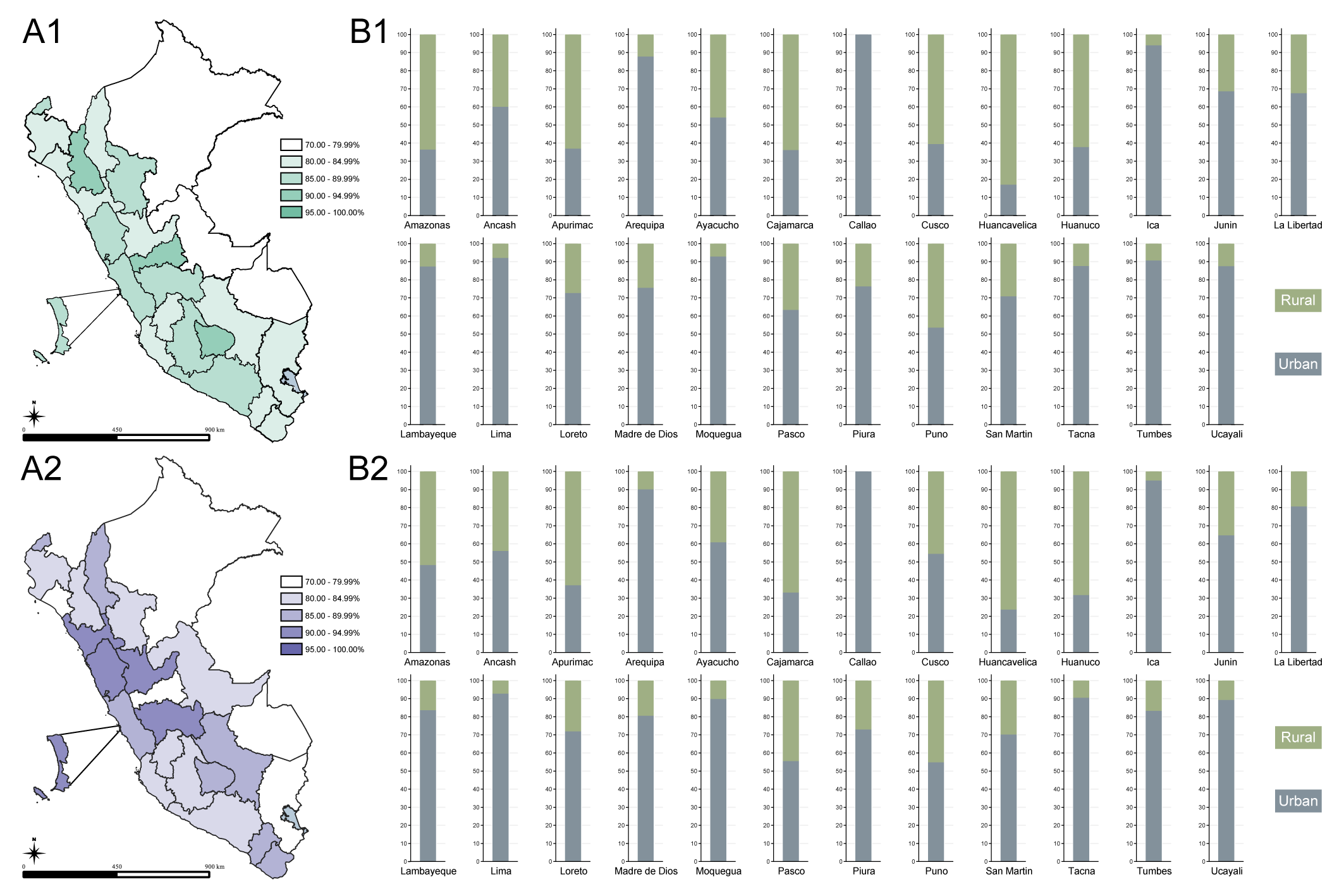

Supplement: S5 Fig — A1: Percentage by region in 2021. B1: Percentage by place of residence in 2021. A2: Percentage by region in 2022. B2: Percentage by place of residence in 2022. Departmental boundaries obtained from official shapefiles of the Instituto Nacional de Estadística e Informática (INEI), accessed via GeoGPS Perú (https://www.geogpsperu.com/2019/08/limite-departamental-politico-shapefile.html). Licensed under the Open Data Commons Attribution License (https://datosabiertos.gob.pe/dataset/limites-departamentales), compatible with CC BY 4.0. (TIF) [file pgph.0005891.s015.tif]

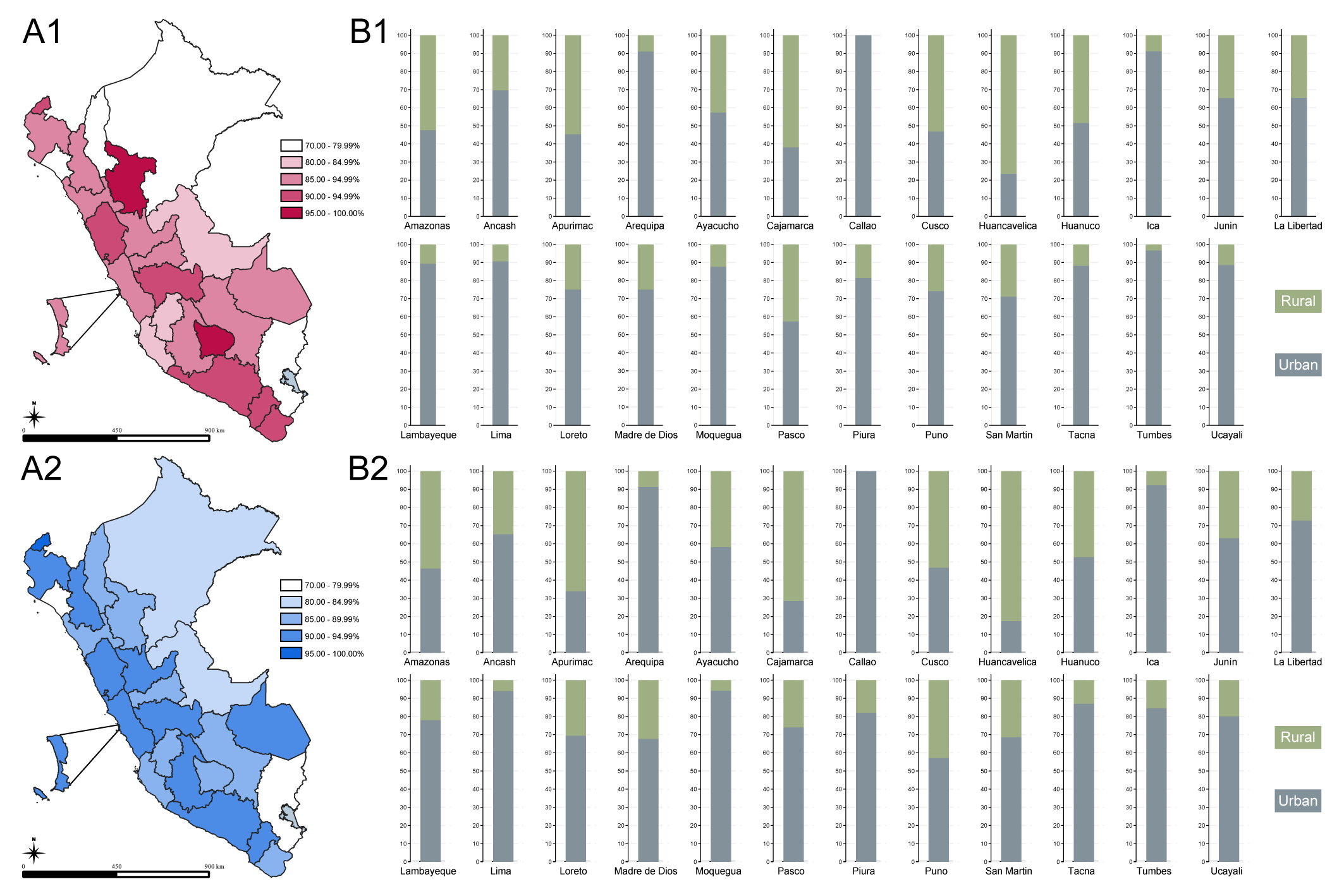

Supplement: S6 Fig — A1: Percentage by region in 2023. B1: Percentage by place of residence in 2023. A2: Percentage by region in 2024. B2: Percentage by place of residence in 2024. Departmental boundaries obtained from official shapefiles of the Instituto Nacional de Estadística e Informática (INEI), accessed via GeoGPS Perú (https://www.geogpsperu.com/2019/08/limite-departamental-politico-shapefile.html). Licensed under the Open Data Commons Attribution License (https://datosabiertos.gob.pe/dataset/limites-departamentales), compatible with CC BY 4.0. (TIF) [file pgph.0005891.s016.tif]

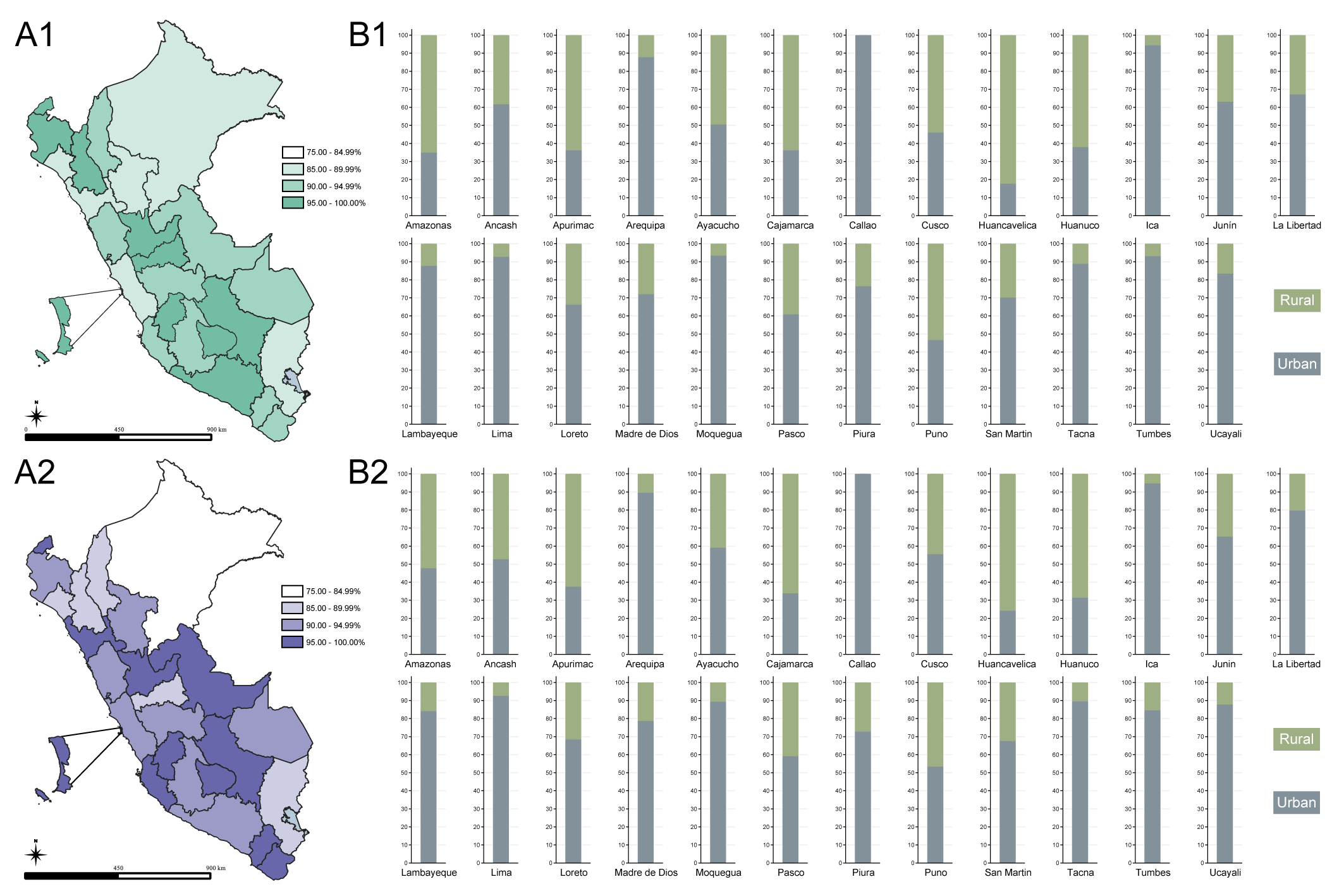

Supplement: S7 Fig — A1: Percentage by region in 2021. B1: Percentage by place of residence in 2021. A2: Percentage by region in 2022. B2: Percentage by place of residence in 2022. Departmental boundaries obtained from official shapefiles of the Instituto Nacional de Estadística e Informática (INEI), accessed via GeoGPS Perú (https://www.geogpsperu.com/2019/08/limite-departamental-politico-shapefile.html). Licensed under the Open Data Commons Attribution License (https://datosabiertos.gob.pe/dataset/limites-departamentales), compatible with CC BY 4.0. (TIF) [file pgph.0005891.s017.tif]

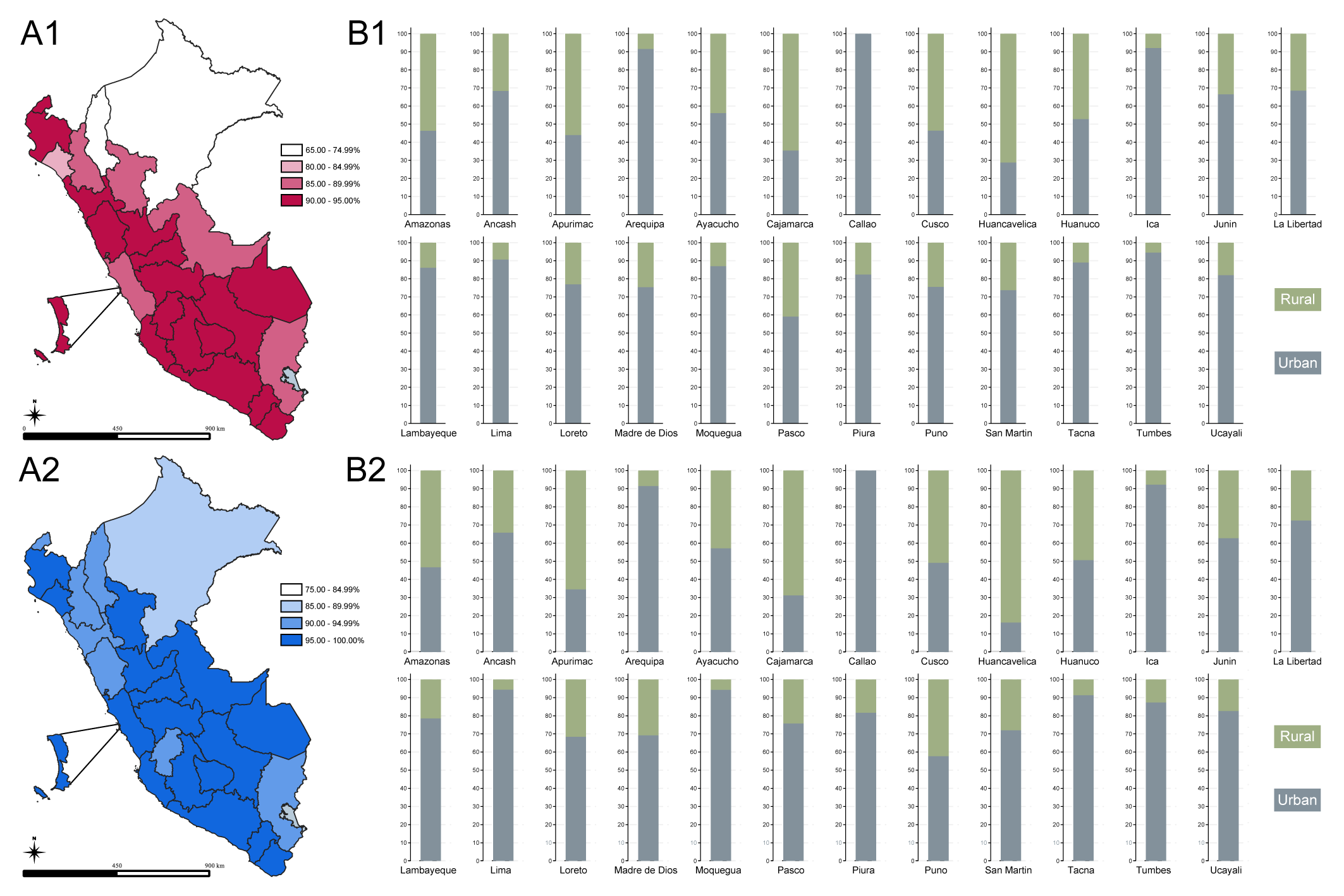

Supplement: S8 Fig — A1: Percentage by region in 2023. B1: Percentage by place of residence in 2023. A2: Percentage by region in 2024. B2: Percentage by place of residence in 2024. Departmental boundaries obtained from official shapefiles of the Instituto Nacional de Estadística e Informática (INEI), accessed via GeoGPS Perú (https://www.geogpsperu.com/2019/08/limite-departamental-politico-shapefile.html). Licensed under the Open Data Commons Attribution License (https://datosabiertos.gob.pe/dataset/limites-departamentales), compatible with CC BY 4.0. (TIF) [file pgph.0005891.s018.tif]
